# Targeting of CD122 enhances antitumor immunity by altering the tumor immune environment

**DOI:** 10.18632/oncotarget.22642

**Published:** 2017-11-24

**Authors:** Daniel O. Villarreal, Michael J. Allegrezza, Melissa A. Smith, Diana Chin, Leopoldo L. Luistro, Linda A. Snyder

**Affiliations:** ^1^ Oncology Discovery, Janssen R&D, Spring House, PA 19477

**Keywords:** CD122, vaccines, GITR, immunotherapy, CD8 T cells

## Abstract

Mounting evidence demonstrates that CD8^+^CD122^+^ T cells have suppressive properties with the capacity to inhibit T cell responses. Therefore, these cells are rational targets for cancer immunotherapy. Here, we demonstrate that CD122 monoclonal antibody (mAb; aCD122) therapy significantly suppressed tumor growth and improved long-term survival in tumor-bearing mice. This therapeutic effect correlated with enhanced polyfunctional, cytolytic intratumoral CD8^+^ T cells and a decrease in granulocytic myeloid-derived suppressor cells (G-MDSCs). In addition, aCD122 treatment synergized with a vaccine to augment vaccine-induced antigen (Ag)-specific CD8^+^ T cell responses, reject established tumors and generate memory T cells. Furthermore, aCD122 mAb synergized with an anti-GITR (aGITR) mAb to confer significant control of tumor growth. These results suggest CD122 might be a promising target for cancer immunotherapy, either as a single agent or in combination with other forms of immunotherapy.

## INTRODUCTION

Tumors use an array of immunosuppressive mechanisms to attenuate tumor-reactive immune responses [1]. CD4^+^ regulatory T cells (Tregs) and myeloid-derived suppressor cells (MDSCs) are two of the major cell types that can promote tumor development and progression by suppressing effective antitumor immunity [2-4]. Recently, a subset of CD8^+^ T cells, CD8^+^CD122^+^, has been identified to have suppressive properties. Studies have shown that CD8^+^CD122^+^ T cells are involved in maintaining T-cell homeostasis and suppressing the proliferation and IFNγ production of T cells [5-8]. It is possible that CD8^+^CD122^+^ suppressive T cells may contribute to the inhibition of antitumor immunity. CD8^+^CD122^+^ T cells may be rational targets for immunotherapy, but the anti-tumor effect of targeting CD122 *in vivo* remains to be determined.

Given that tumors take advantage of regulatory suppressive T cells to help them evade immune attacks, it will be important to identify strategies for effectively removing or targeting CD8^+^CD122^+^ suppressive T cells. Thus, we investigated whether targeting the CD8^+^CD122^+^ T cell subpopulation with an anti-CD122 (aCD122) monoclonal antibody (mAb) would enhance antitumor immunity. We show that aCD122 therapy enhanced T cell-mediated tumor rejection and reduced intratumoral G-MDSCs. In addition, our study supports the pairing of CD122 targeting therapies with cancer vaccines or CD4^+^ Treg-depleting modalities for immunotherapy.

## RESULTS AND DISCUSSION

### CD122 mAb treatment impairs tumor growth in syngeneic solid tumor models

**Figure 1:**
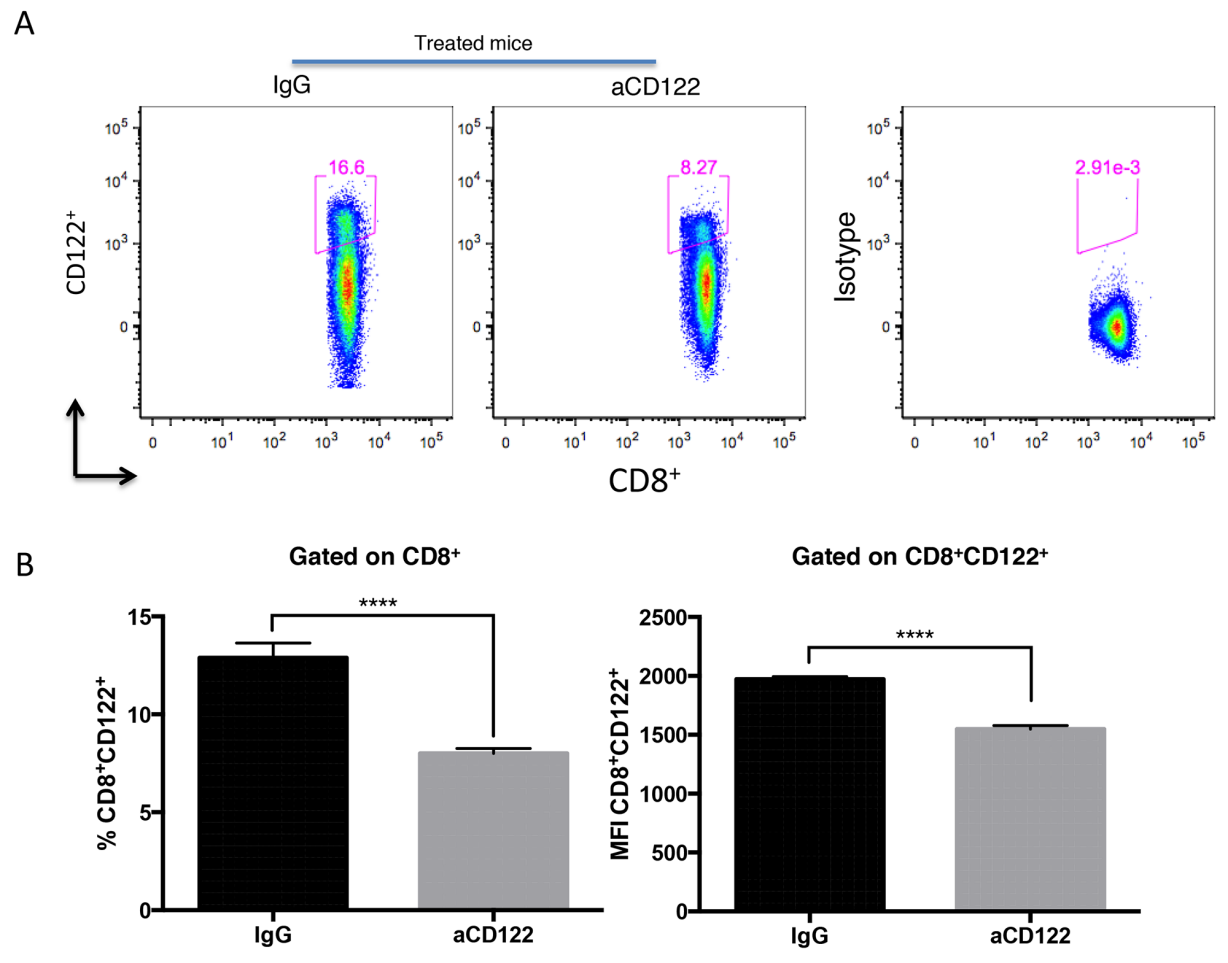
Treatment with 5H4 mAb reduced CD8^+^CD122^+^ T cell population Naïve B6 non-tumor bearing mice were injected i.p. with 100 ug of 5H4 anti-CD122 mAb or with the isotype (rat IgG2a) control. Three days after injection, splenocytes were analyzed for CD8^+^CD122^+^ T cells by flow cytometry. **(A-B)** Flow plot analysis and representative data showing the percentages of CD8^+^CD122^+^ T cells and the MFI of CD122 on CD8^+^CD122^+^ T cells in total splenic lymphocytes. Isotype indicates CD8^+^ splenocytes stained with the isotype control antibody for CD122 staining. Results are representative of 2 independent experiments. ^****^P<0.0001. Errors bars indicate SEM of n = 5/group.

Given that CD8 T cell suppressor cells (CD8^+^CD122^+^ in mice) have been shown to blunt Ag-specific T cell responses [5-7], we hypothesized that targeting murine CD8^+^CD122^+^ T cells with an aCD122 mAb would enhance antitumor immune responses. We first examined if the aCD122 mAb antibody (clone 5H4) could reduce the CD8^+^CD122^+^ T cell target population. The 5H4 clone was selected on the basis that 5H4 antibody does not inhibit binding of IL-2 to the IL-2R complex (of which CD122 is a member) [21]. To detect CD122 expression on CD8^+^ T cells, we used a different aCD122 antibody (clone TM-β1) that recognizes an alternative noncompeting epitope from 5H4 [9-10]. Our data showed that the population of CD8^+^CD122^+^ T cells in the spleen was significantly depleted ∼30-40%, 3 days after aCD122 (5H4) treatment (Figure 1A-1B).

**Figure 2:**
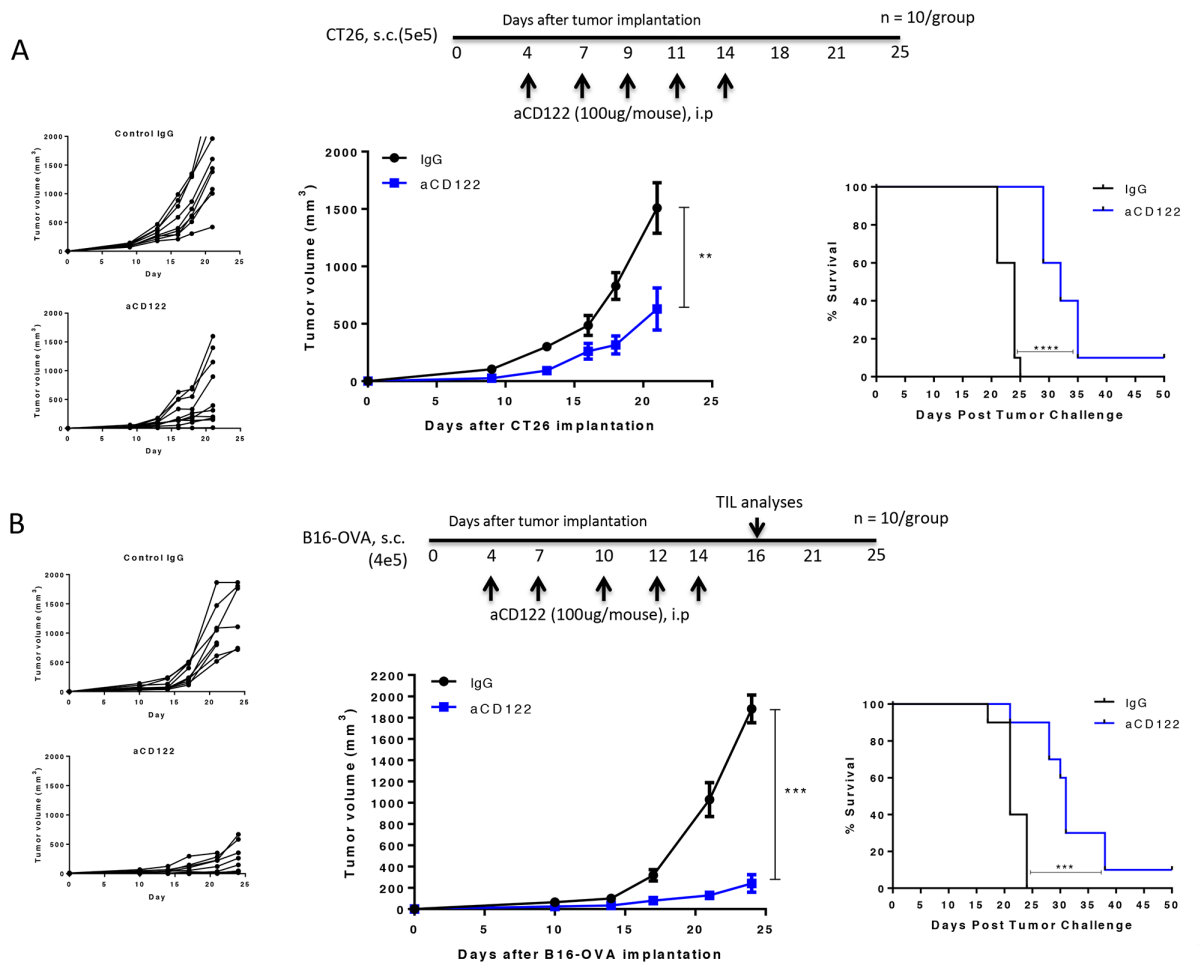
CD122 mAb treatment suppressed CT26 and B16-OVA tumor growth and enhanced long-term survival Treatment regimen, individual tumor growth, group tumor measurements, and survival of CT26 **(A)** and B16-OVA **(B)** implanted mice following treatment are indicated. Figures represent 2 (CT26) and 3 (B16-OVA) independent experiments. ^**^P<0.01; ^***^P<0.001; ^****^P<0.0001.

We next examined the impact of aCD122 treatment in mice bearing CT26 colon carcinoma and B16-OVA melanoma tumors. We found that treatment with aCD122 significantly suppressed tumor growth in the CT26 model (Figure 2A). CD122 mAb treatment enhanced long-term survival with 10% of the mice rejecting tumors, even after aCD122 treatment was discontinued. Similarly, we found that treatment with aCD122 showed significant B16-OVA tumor growth inhibition and long-term survival (Figure 2B). These results demonstrate that aCD122 treatment can control the growth of solid tumors and enhance overall survival.

### CD122 mAb treatment alters the tumor immune microenvironment

**Figure 3:**
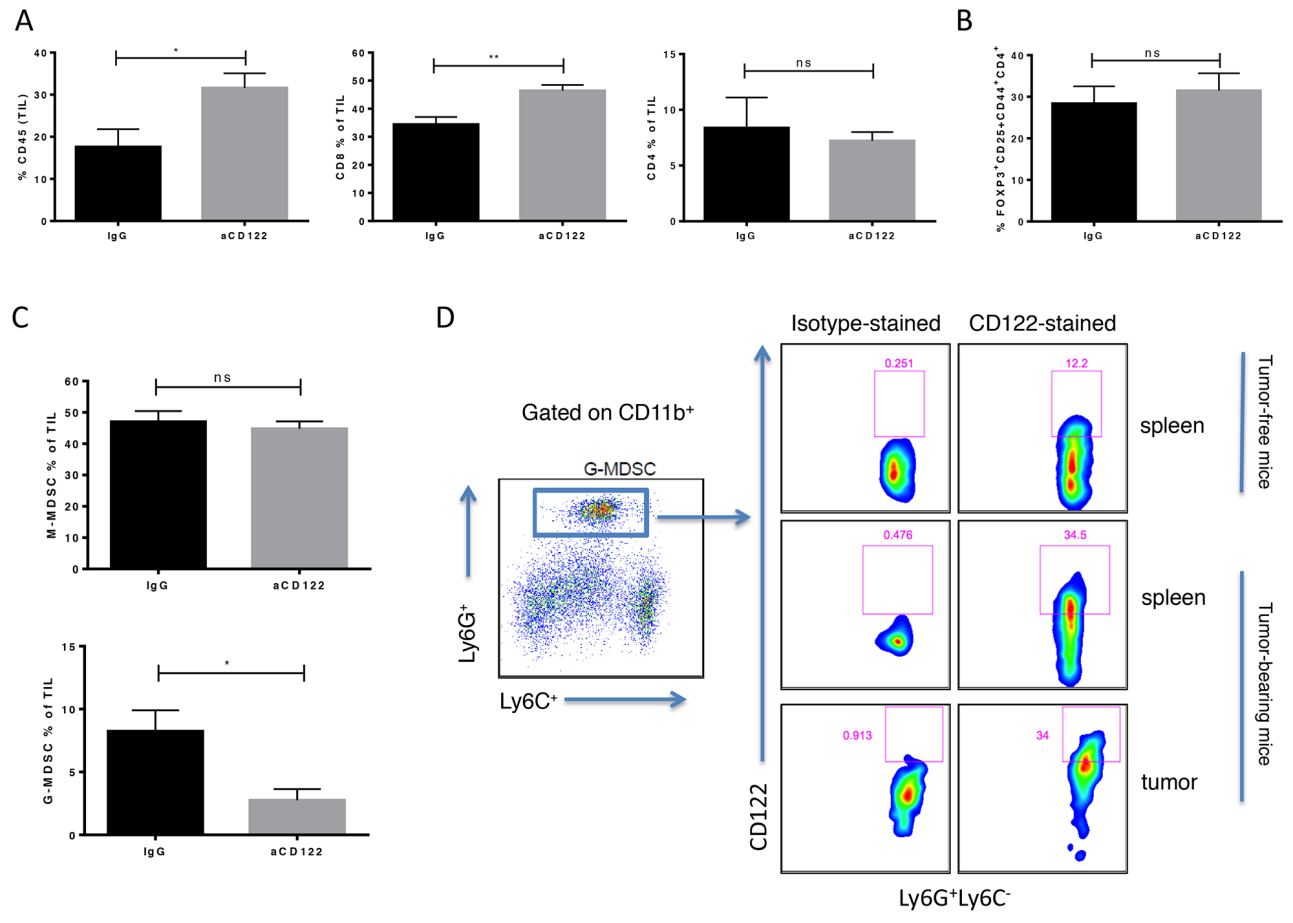
CD122 mAb treatment altered the cellular composition of the tumor immune microenvironment in the B16-OVA melanoma model TILs from tumors of B16-OVA mice were harvested 16 days after tumor implantation. **(A)** CD45^+^ leukocyte infiltrate and CD8^+^ and CD4^+^ TILs as percentage of total CD45^+^ cells are shown in treated versus untreated groups. TIL populations, including CD4^+^ Tregs **(B)** and M-MDSC (CD11b^+^Ly6C^+^Ly6G^-^) and G-MDSC (CD11b^+^Ly6C^-^Ly6G^+^) **(C)** were identified by flow cytometry. **(D)** CD122 expression by G-MDSC was shown by flow cytometry. M-MDSCs also expressed CD122 (data not shown). ^*^P<0.05; ^**^P<0.01; ns not significant. Error bars indicate SEM of n = 5/group.

We next investigated the changes in the tumor microenvironment (TME) induced by aCD122 treatment using the B16-OVA tumor model. CD122 mAb treatment altered the tumor-infiltrating lymphocytes (TIL) composition in the B16-OVA model (Figure 3). Mice treated with aCD122 showed significantly increased infiltration of total CD45^+^ cells in the tumor compared with control treated mice (Figure 3A). Within the TIL CD45^+^ population, aCD122 treatment did not affect the CD4^+^ T cell or CD4^+^ Treg (Foxp3^+^CD25^+^CD44^+^CD4^+^) levels (Figure 3A-3B), a finding that aligns with previous studies in non-malignant diseases reporting that these cell populations are not modulated by aCD122 treatment [9-11]. These results suggest that aCD122 limits tumor growth by a mechanism that does not significantly involve CD4^+^ Tregs. However, treatment with aCD122 did profoundly increase the percentages of tumor-infiltrating CD8^+^ T cells (Figure 3A), and reduced the percentage of infiltrating CD8^+^CD122^+^ T cells (Supplementary Figure 2D). Another population of cells, MDSCs, is capable of suppressing immune responses against tumors [4]. Recent studies have demonstrated a preferential accumulation of G-MDSC over M-MDSC in murine tumor models and in human cancer patients [12-14]. Interestingly, CD122 mAb treatment in B16-OVA tumors significantly reduced the frequency of the G-MDSC (CD11b^+^Ly6C^-^Ly6G^+^) population, but did not significantly alter the M-MDSC (CD11b^+^Ly6C^+^Ly6G^-^) population (Figure 3C). We next determined if the G-MDSCs expressed CD122. Our data demonstrates that CD122 is expressed on splenic G-MDSCs of non-tumor-bearing mice and upregulated in splenic tumor-treated mice and on tumor-infiltrating G-MDSCs (Figure 3D). This expression on G-MDSCs suggests that aCD122 therapy could potentially modulate such populations directly. Thus, aCD122 mAb treatment can alter the TME by enhancing the frequency of tumor-infiltrating CD8^+^ effector T cells and reducing the frequency of G-MDSCs.

### CD122 mAb increases TIL activation and antigen-specific inflammatory cytokine production and is CD8 T cell dependent

**Figure 4:**
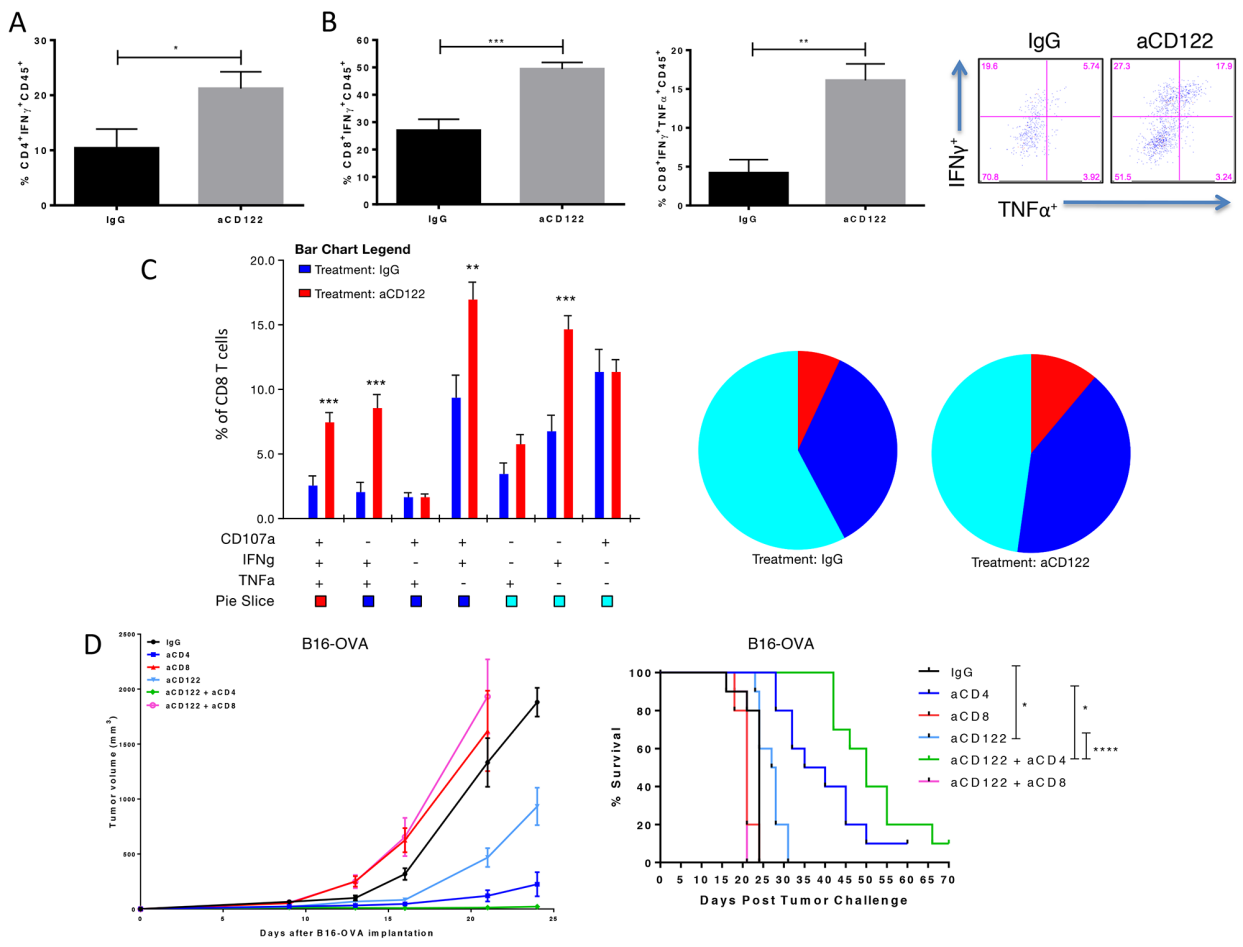
CD122 mAb treatment of B16-OVA tumors increased the frequency and function of specific inflammatory cytokine production of tumor infiltrating T cells and efficacy is dependent on CD8^+^ T cells **(A)** Summary data showing percentage of CD4^+^CD45^+^ TILs expressing IFNγ. **(B-C)** Representative and summary data showing single-, double- and triple positive CD8^+^ T cells releasing IFNγ, TNFα and/or co-expressing the degranulation marker, CD107a, following OVA_257-264_ and OVA_323-339_ peptide incubation with PMA/ION stimulation. **(D)** Tumor growth curves and survival over time for B16-OVA tumor-bearing mice treated aCD122 and with/without anti-CD4 or anti-CD8 mAbs. Experiments were repeated at least two times. ^*^P<0.05; ^**^P<0.01; ^***^P<0.001. Errors bars indicate SEM of n = 5/group (A-C); n= 10/group in (D).

Given the significant changes in the TME, we next characterized the functional capacity of tumor-reactive TILs following aCD122 therapy. Total leukocytes were isolated from tumors 16 days after tumor implantation and stimulated with PMA/ION containing CD4- and CD8-restricted peptides of ovalbumin. TIL cultures derived from mice treated with aCD122 showed significant increases in the frequency of IFNγ positive effector CD4^+^ and CD8^+^ T cells compared to the control group (Figure 4A-4B). In addition, aCD122 therapy significantly increased the frequency of IFNγ/TNFα dual-positive CD8^+^ T cells within the tumor (Figure 4B), as well as polyfunctional effector CD8^+^ T cells coexpressing CD107a/IFNγ/TNFα, compared to control group (Figure 4C). These results indicate the potential of aCD122 therapy to enhance functional effector cytolytic T cells, which may have greater potential to kill tumor cells.

We next investigated the relevance of the effector populations on tumor rejection induced by the aCD122 therapy. In the B16-OVA model, tumor-bearing mice were treated with aCD122 and depleted of CD8^+^ T cells, CD4^+^ T cells, and NK cells to determine their role in sustaining tumor protection mediated by aCD122. Treatment with anti-CD8 mAb completely abrogated the beneficial effects provided by aCD122 treatment, as no mice survived past 21 days post-tumor implantation (Figure 4D). In contrast, the depletion of CD4^+^ T cells did not inhibit the antitumor activity of aCD122 therapy, suggesting that aCD122 treatment can act independently of helper CD4^+^ T cells and CD4^+^ Tregs. In addition, depletion of NK cells had no affect on the antitumor activity of aCD122 therapy (Supplementary Figure 1A), indicating these cells played no role in the efficacy observed. The data supports the conclusion that CD8^+^ T cells are critical for the tumor protection observed by CD122 therapy. Moreover, in accordance with previous studies, we observed that CD4 depletion alone prolonged survival, likely due to the removal of all CD4^+^ Tregs [15-16]. Interestingly, we observed dramatic benefit of administering aCD4 with aCD122 treatment, improving tumor control and overall survival (Figure 4D). This finding raised the intriguing possibility that the modulation/depletion of CD8^+^CD122^+^ T cells and CD4^+^ Tregs could be complementary strategies to achieve tumor control.

**Figure 5:**
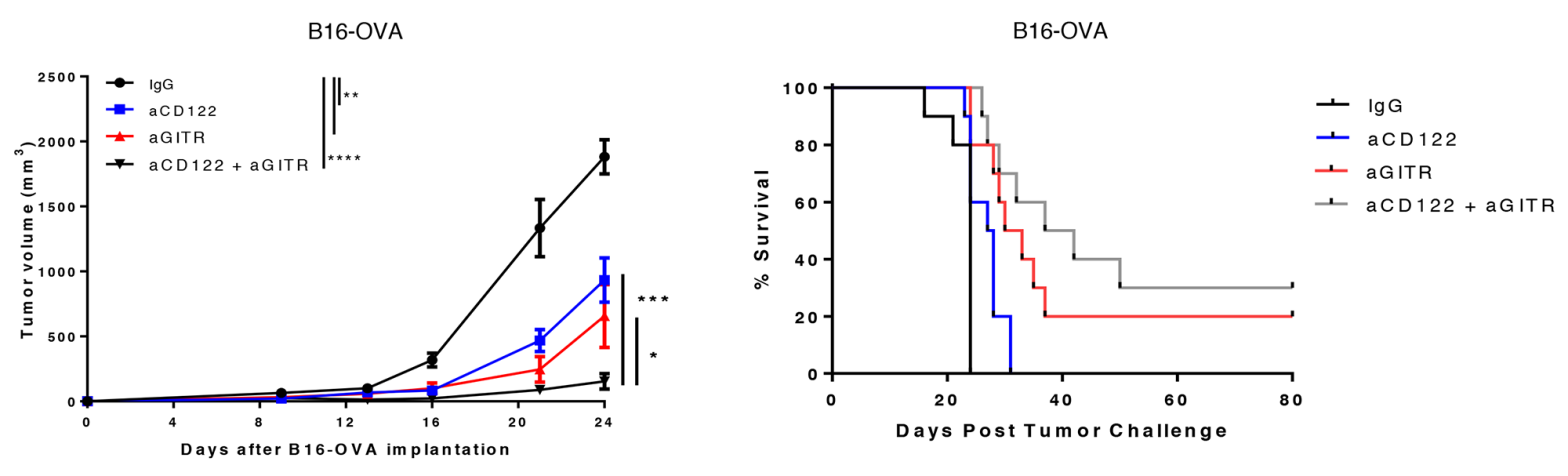
Anti-CD122 mAb treatment synergizes with an aGITR mAb therapy On day +4 post-implantation of B16-OVA, mice were treated with aCD122 or aGITR or the combination as indicated. Mean tumor growth and survival are depicted. Experiments were repeated at least two times. ^*^P<0.05; ^**^P<0.01; ^***^P<0.001; ^****^P<0.0001. Errors bars indicate SEM; n = 10/group.

To test this hypothesis, we first investigated the effects of depleting Tregs by administering aCD25 mAb along with aCD122 therapy. We observed no additive benefit of administering aCD25 with aCD122 therapy (Supplementary Figure 1B). This observation may be due to the accompanying depletion by aCD25 of some effector CD4^+^ and CD8^+^ T cells that upregulate CD25 following activation [17-18]. We therefore sought to combine aCD122 with an alternative immunotherapy known to modulate Tregs, aGITR. GITR mAb is an immunotherapy capable of specifically reducing the number of CD4^+^ Tregs in the tumor [19-20]. Therapeutic intervention on 4-day B16-OVA tumors using aCD122 and aGITR targeting mAbs demonstrated an additive effect, showing significant suppression of tumor growth that yielded 30% long-term survival, compared to aCD122 monotherapy (Figure 5). These results demonstrate that aGITR in combination with aCD122 therapy can lead to greater tumor growth control. We speculate that the ability of aCD122 to reduce suppressive CD8^+^CD122^+^ T cells/G-MDSCs and the ability of aGITR to reduce CD4^+^ Tregs and promote T cell function contributed to the enhancement of tumor growth control.

### aCD122 treatment synergizes with a tumor vaccine to achieve optimal therapeutic efficacy

**Figure 6:**
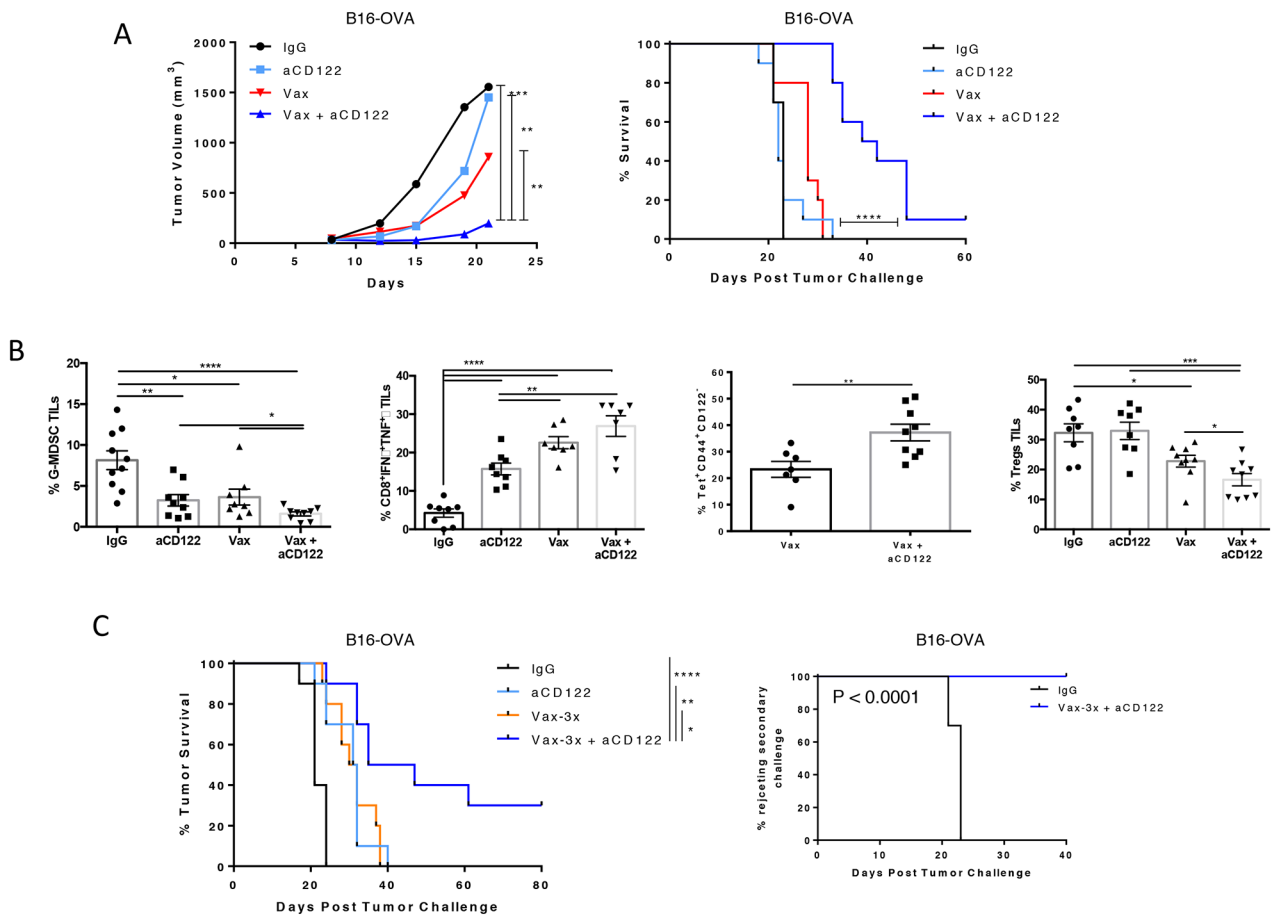
CD122 mAb synergized with a tumor vaccine to inhibit the growth of B16-OVA tumors **(A)** On day +7 post-implantation, mice were treated with aCD122 mAb (administered 5 times at 2-3 day intervals) or with peptide vaccine (1 dose) or the combination as indicated. Mean tumor growth and survival are depicted **(B)**. TILs were harvested at day +16, followed by analysis of percent of tumor infiltrating G-MDSCs, IFNγ/TNFα cytokine secreting CD8^+^ T cells upon *ex-vivo* stimulation with OVA_257-264_ peptide, OVA tetramer-specific CD8^+^ TILs, and Tregs. **(C)** On day +7 post-implantation, mice were treated with aCD122 in combination with peptide vaccine (3 doses) as indicated. Survival curve on the left shows primary treatment results; curve on the right shows rechallenged mice that rejected tumors after aCD122/vaccine treatment. Data are representative of 2-3 independent experiments. ^*^P<0.05; ^**^P<0.01; ^***^P<0.001; ^****^P<0.0001. Error bars indicate SEM of n = 5/group for the bar graphs; n=10/group for the efficacy studies in B, and D.

Although aCD122 as a monotherapy delayed tumor progression, it was not curative in a more stringent therapeutic intervention on 7-day tumors under the conditions tested (Figure 6A). Therefore, in order to enhance the magnitude of the tumor-specific immune response, a peptide-based cancer vaccine targeting the OVA neo-tumor antigen (SIINFEKL) was used in combination with aCD122 therapy. Therapeutic intervention of 7-day established tumors (with average tumor diameter ∼30-40 mm^3^) using aCD122 and a single dose of peptide vaccine showed significant suppression of tumor growth, leading to ∼10% long-term survival, whereas either monotherapy had little to no effect (Figure 6A). Analysis of tumor-infiltrating leukocytes showed that when aCD122 was combined with the vaccine, there was a significant reduction in the frequency of G-MDSCs relative to each agent alone (Figure 6B). In addition, combination therapy significantly increased the dual IFNγ/TNFα production from effector CD8^+^ TILs and enhanced the OVA-tetramer-specific CD8^+^CD44^+^ memory T cells in the tumors (Figure 6B). The increase of OVA-tetramer-specific CD8^+^ T cells was also noted in the periphery of non-tumor bearing mice treated with the combination of vaccine and aCD122 therapy (Supplementary Figure 2A-2B). Importantly, the combination therapy markedly reduced the frequency of CD4^+^ Tregs relative to each agent alone (Figure 6B), suggesting the overall improved protection observed in the combination group was associated with (1) increased Ag-specific CD8^+^ T cell responses, (2) decreased G-MDSCs and (3) reduction of CD4^+^ Treg populations in the tumor. These changes are consistent with an immune-environment that favors tumor rejection.

Finally, a prime-boost vaccination strategy was applied on day +7, +10, and +14 for treating 7-day established tumors and showed greater long-term survival when combined with aCD122 (30%) than a single vaccine dose in this therapeutic setting (Figure 6C). Notably, tumor rejection occurred even though aCD122 treatment was stopped after day 17, indicating that prime-boost vaccination plus temporary aCD122 can completely overcome tumor-driven immunosuppression in some mice. Given that CD8^+^CD122^+^ T cells have been described to have memory CD8^+^ T-cell properties [5], we examined if targeting such a population affected the generation of long-lasting memory T cells. A second tumor challenge was performed using the tumor-free prime-boost survivors at day +80 post-treatment. The results showed that these mice remained protected from tumor growth, indicating that T-cell antitumor memory was developed and maintained by the combination therapy (Figure 6C).

## MATERIALS AND METHODS

### Mice and tumor cells

Female, 6-8 weeks old C57BL/6 (B6) and Balb/c mice were purchased from Jackson Laboratories. All mouse procedures were performed in accordance with protocols approved by the Janssen Pharmaceuticals IACUC (Spring House, PA). The B16F10-OVA (B16-OVA) cell line was obtained from K. Rock (University of Massachusetts Medical School). The CT26.WT (CRL-2638) mouse colon carcinoma cell line was purchased from ATCC.

CT26 cells were maintained in RPMI media supplemented with 10% FBS in a humidified atmosphere with 5% CO_2_ at 37°C. The B16-OVA cell lines were maintained in Hybridoma culture media (HCM). HCM contains the following components: 10% FCS, 1% penicillin/streptomycin, 1% L-glutamine, 1% NEAA (non-essential amino acids), 1% HEPES and 1M 2-βME. All cell lines were authenticated and determined to be free of *Mycoplasma*.

### Tumor models, tumor vaccine, and treatments

B16-OVA (400,000 for challenge and 200,000 for rechallenge) and CT26 (500,000) cells were implanted subcutaneously (s.c.) in the right flank of mice. Tumor vaccine consisted of poly (I:C) (100 μg; InvivoGen), CpG (ODN1826, 30 μg; InvivoGen), OVA CD4-helper (ISQAVHAAHAEINEAGR) peptide (323-339, 20 μg) and OVA CD8-restricited (SIINFEKL) peptide (257-264, 20 μg) (MBL International) all mixed in PBS. Mice were immunized with 200 μl of vaccine mixture s.c. on indicated days. For therapeutic treatments, mice were treated with intraperitoneal (i.p.) injections of anti-CD122 targeting mAb (aCD122, clone 5H4 rat IgG_2a_; 100 μg/mouse/injection), anti-GITR targeting mAb (aGITR, clone DTA-1 rat IgG_2b_, 200 μg/mouse/injection) and control antibodies (rat IgG_2a_, Clone 2A3; rat IgG_2b_, Clone LTF-2; mouse IgG_2a_, Clone C1.18.4; rat IgG_1_, Clone HRPN), all purchased from BioXcell. For *in vivo* cell depletion, anti-CD4 mAb (clone GK1.5, rat IgG_2b_ 0.2 mg/dose), anti-CD8 mAb (clone 53-6.72 rat IgG_2a_ 0.2 mg/dose), anti-CD25 (clone PC-61.5.3 rat IgG_1_ 0.2 mg/dose) and aNK1.1 mAb (clone PK136, mouse IgG2a 0.2 mg/dose) were injected i.p. on indicated days. Tumor growth was monitored using electronic calipers and calculated according to the formula: V = (length x width^2^)/2.

For *in vivo* cell depletion in Figure 5A, mice were injected s.c. with 4x10^5^ B16-OVA tumor cells and were treated with either aCD4, aCD8, aCD25, and aNK1.1 mAb’s, i.p. injection, on days +3, +4, +5, +8, +11, +14, with day +4 as the day when aCD122 or control IgG treatment started.

For therapeutic anti-CD122 mAb treatment in Figure 6A, mice were treated with i.p. injection of 100 μg mAb on days +7, +9, +12, +14, and +16 post tumor inoculation.

Mice were vaccinated s.c. either once on day +7 (Figure 6B) or primed/boosted on day +7, +10, and +14 (Figure 6D). For single vaccination, aCD122 was administered as mentioned above, while for the prime/boost experiment, aCD122 was administered on day +7, +10, +12, +14 and +17. To analyze tumor-infiltrating T cells upon vaccine/anti-CD122 combination treatment, tumors from all groups were harvest for analysis around 1 week post treatment.

For therapeutic anti-GITR and anti-CD122 combination treatment in Figure 5A, mice were treated i.p. with 200 μg of aGITR mAb on days +4 and +10 post tumor implantation and with 100 μg of aCD122 mAb i.p. on days +4, +7, +9, +11 and +14 post-tumor implantation.

### Leukocyte Isolation

TIL isolation: In Figure 3-4, tumors were harvested around 16 days after tumor implantation. Tumors were digested with 5ml of 1mg/ml collagenase IV (Stem Cell Technologies) for 30 minutes at 37°C. After incubation, TILs were isolated by mechanical disruption of the tumor using a Stomacher machine. The resulting product was filtered using a 70 μm cell strainer. Cells were pelleted and then resuspended in RPMI medium into 96-well plates for use in flow cytometry assays as described below.

Splenocyte Isolation: Spleens were collected in RPMI 1640 medium supplemented with 10% FBS, 1× penicillin/streptomycin, and 1× β-ME. Splenocytes were isolated by mechanical disruption of the spleen using a Stomacher machine (Seward Laboratory Systems). The resulting mashed spleens were filtered using a 40 μm cell strainer, pelleted, and treated with ACK lysis buffer for 5 minutes to lyse the red blood cells, washed in PBS and then resuspended in RPMI medium for use in flow cytometry assays.

Lymphocyte isolation from blood: Peripheral blood was collected in 500 ul of 4% sodium citrate. The suspension was under-laid with 2ml of Histopaque-1083 (Sigma) and spun at 2000 rpm for 20 min at 20°C (Speed: Acceleration 3, Deceleration 0). Lymphocytes were harvested from the interface and washed once in RPMI and resuspended into 96 well plates for use in flow cytometry assays.

### Flow cytometry

Splenocytes or TILs were added to a 96-well plate (1 x 10^6^ cells/well), cells were incubated for 30 min at 4°C with antibodies for CD45, CD4, CD8, CD44, CD25, CD122 (5H4 or TM-β1 clone), TCRb, CD11b, Ly6G, Ly6C, LIVE/Dead fixable violet dead cell stain kit, and MHC class I peptide tetramer to H2-K^b^-SIINFEKL-OVA. All antibodies were obtained from eBioscience, BD Biosciences, Biolegend and/or MBL International. Intracellular cytokine staining was performed after 5 hours of *ex vivo* stimulation with 1x Cell Stimulation Cocktail plus protein transport inhibitors (ebioscience), plus 2.5 ug/ml of OVA_257-264_ CD8 peptide (SIINFEKL) and OVA_323-368_ CD4 peptide with or without the CD107a FITC antibody (degranulation marker) for 5 hours. For intracellular staining, cells were fixed and permeabilized with either the Biolegend or FoxP3 staining buffer kit (ebioscience) according to the manufacturer’s instructions. Cells were incubated for 45 min at 4°C with antibodies to IL-2, TNFα, IFNγ, CD3 and FoxP3. Cells were collected and analyzed using the Fortessa flow cytometer using DIVA (BD Biosciences) and analyzed using FlowJo software (Tree Star, Ashland, OR). Boolean gating was performed using FlowJo software to examine the polyfunctionality of the T cells from treated animals and analyzed using SPICE v5.3 (freely available from http://exon.niaid.nih.gov/spice/).

### Statistical analysis

Statistical significance was determined by unpaired Student T test (two-tailed), and for tumor survival analysis, Kaplan-Meier test was used. Error bars indicate standard error of the mean (SEM). All graphs and statistical analysis were generated using Prism 6 software (GraphPad Software, Inc). All data are representative of 2-3 independent experiments.

## CONCLUSION

The immunosuppressive TME, which includes suppressive T cells and MDSCs, represents a major obstacle for effective tumor immunotherapy. Recently, CD8^+^CD122^+^ T cells were reported to have regulatory properties, suppressing autoimmunity and inhibiting T cell responses [5-8]. Here, we provide evidence that targeting CD122 can modulate the adaptive immune response and suppress tumor growth. These *in vivo* antitumor responses were CD8^+^ T cell-dependent and associated with the increase of tumor-reactive cytolytic, polyfunctional CD8^+^ T cells undergoing degranulation. In accordance with previous studies [9-11], we observed that aCD122 therapy did not affect the levels of CD4^+^ Tregs. Interestingly, we found that the improved antitumor activity of targeting CD122 was also associated with the reduction of G-MDSCs in the TME. It is well known that tumor infiltrating myeloid cells, such as G-MDSCs, are involved in suppressing tumor-specific T-cell responses [4]. Thus, given that G-MDSCs can express CD122 (Figure 3D), our data suggests that CD122 therapy may potentially regulate G-MDSCs. However, its role in directly or indirectly modulating the G-MDSC population clearly warrants additional investigation. Furthermore, how aCD122 may potentially affect the immune function of other cell populations requires more study. Nevertheless, this novel strategy of targeting CD122, which can increase CD8^+^ T cells and reduce G-MDSCs, could have a wide range of applications and therefore be effectively combined with other immunotherapies.

We show that reducing CD8^+^CD122^+^ T cells can also modulate the expansion of CD8^+^ T cells in a vaccine setting, thus leading to significant antitumor immunity and tumor regression in established melanoma tumor-bearing mice. In addition, optimal tumor control and rejection in the combination Vax/aCD122 therapy was associated with reduction of both suppressive CD4^+^ Tregs and G-MDSCs in the TME. The enhanced production of Th1 cytokine-producing T cells in the tumor likely shifts the TME from a suppressive to a more inflammatory state, yielding more powerful tumor growth control.

In addition, we demonstrated that aCD122 treatment synergized with aCD4, indicating its efficacy was independent of CD4 helper T cells and the overall improved efficacy was likely due to removal of CD4 Tregs. Combination of aCD122 with a CD25-depleting antibody did not show improved efficacy, which may be from an accompanying depletion by aCD25 of some effector CD4 and CD8 T cells that upregulate CD25 following activation [17-18]. Alternatively, a population of regulatory CD4 T cells that are CD25- (thus not depleted by aCD25) could play a role in this tumor model. IL-10 producing type-1 regulatory T cells (Tr1), characterized as CD4^+^Foxp3^-^CD25^-^CD44^hi^ could fit this description. Tr1 cells have been reported to express high-levels of GITR [22]. We have demonstrated previously, along with others that GITR-triggering with a GITR mAb can enhance T cells via costimulation and reduce the number of CD4 Tregs in the tumor [19-20]. Although we did not analyze Tr1 cells following aGITR treatment, it is possible that they were reduced by aGITR, but unaffected by aCD25. Because we observed that aCD122 treatment synergized with an aGITR, but not aCD25, Tr1 cells may contribute to suppress antitumor immunity in addition to CD8^+^CD122^+^ T cells in our experiments. However, further studies are needed to elucidate these mechanisms. Surprisingly, we observed that aCD122 treatment synergized with an aGITR mAb to improve tumor efficacy. We speculate that both the reduction of tumor infiltrating regulatory CD4 T cells and costimulation of effector T cells by aGITR contributed the enhancement of active immunization with aCD122 therapy; additional studies will be required to address this. Collectively, this highlights that development of effective combinatorial strategies can be essential to target the many mechanisms of tumor-induced T cell immunosuppression.

Paradoxically, CD8^+^CD122^+^ T cells have been described as or associated with Ag-specific memory T cells [5]. Cohen et al, showed that a vaccine regime combined with aGITR could induce a high proportion of antigen-specific memory CD8^+^CD122^+^ T cells [17]. However, despite this increase, it did not ultimately translate into improved long-term survival, perhaps because the CD8^+^CD122^+^ T cells can possess suppressive properties. In contrast, we demonstrated that by reducing the CD8^+^CD122^+^ T cell population during combination Vax/aCD122 (Supplementary Figure 2), we could significantly improve long-term tumor-free survival from initial tumor challenge. In addition, we showed that mice that benefited from Vax/aCD122 therapy, when rechallenged, remained completely protected against the same tumor, indicating the establishment of long-lasting memory responses. These results show that reducing the suppressive CD8^+^CD122^+^ T cell population can retain Ag-specific effector memory CD8 T-cell function and ultimately lead to long-term survival.

To the best of our knowledge, this is the first proof-of-concept study illustrating enhancement and persistence of antitumor responses using aCD122 alone or in combination with a vaccine or aGITR mAb immunotherapy. These results establish the validity of CD122 as a target for monotherapy or in combination with additional immune-targeted therapies for treatment of solid tumors, such as melanoma and certain colorectal cancers. In addition, these results establish the conceptual basis for targeting CD8^+^ Tregs and warrant investigations into CD8^+^ Tregs in human cancer.

## SUPPLEMENTARY MATERIALS FIGURES


