# Supplementary material for: Targeting of CD122 enhances antitumor immunity by altering the tumor immune environment
Source: Oncotarget. 2017 Nov 24;8(65):109151–60. doi: 10.18632/oncotarget.22642 (PMC5752510; doi:10.18632/oncotarget.22642)
Supplement: Supplementary file 1 [file oncotarget-08-109151-s001.pdf]

Targeting of CD122 enhances antitumor immunity by altering the tumor immune environment

SUPPLEMENTARY MATERIALS

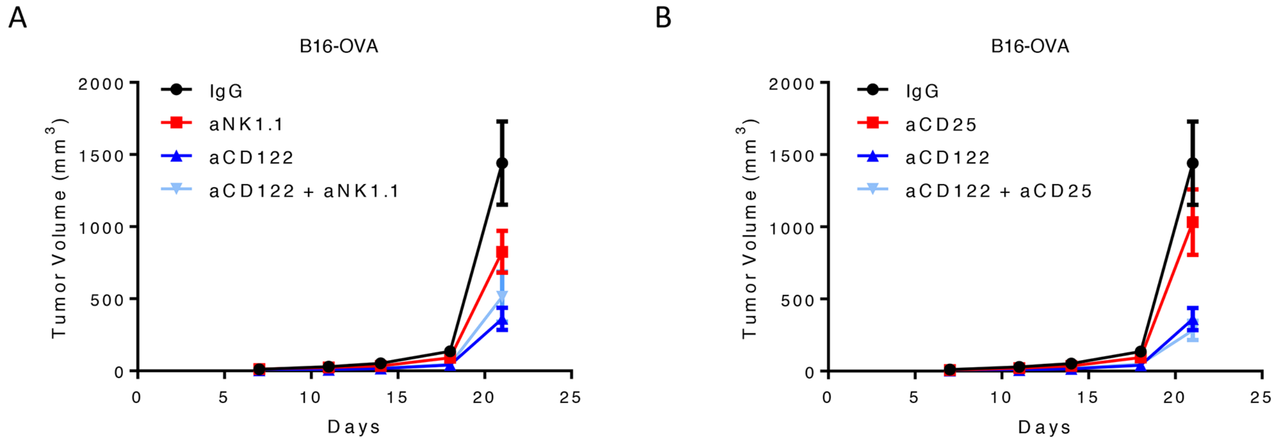

Supplementary Figure 1: Depletion of NK1.1^{+} cells and CD25+ cells with aCD122 mAb therapy does not abrogate or enhance tumor efficacy. (A-B) Tumor growth curves for different groups of B16-OVA tumor-bearing mice treated with or without 200 µg of aNK1.1 or aCD25 mAbs. Mean tumor growth and survival are depicted. Experiments were repeated at least two times. Errors bars indicate SEM; n = 10/group.

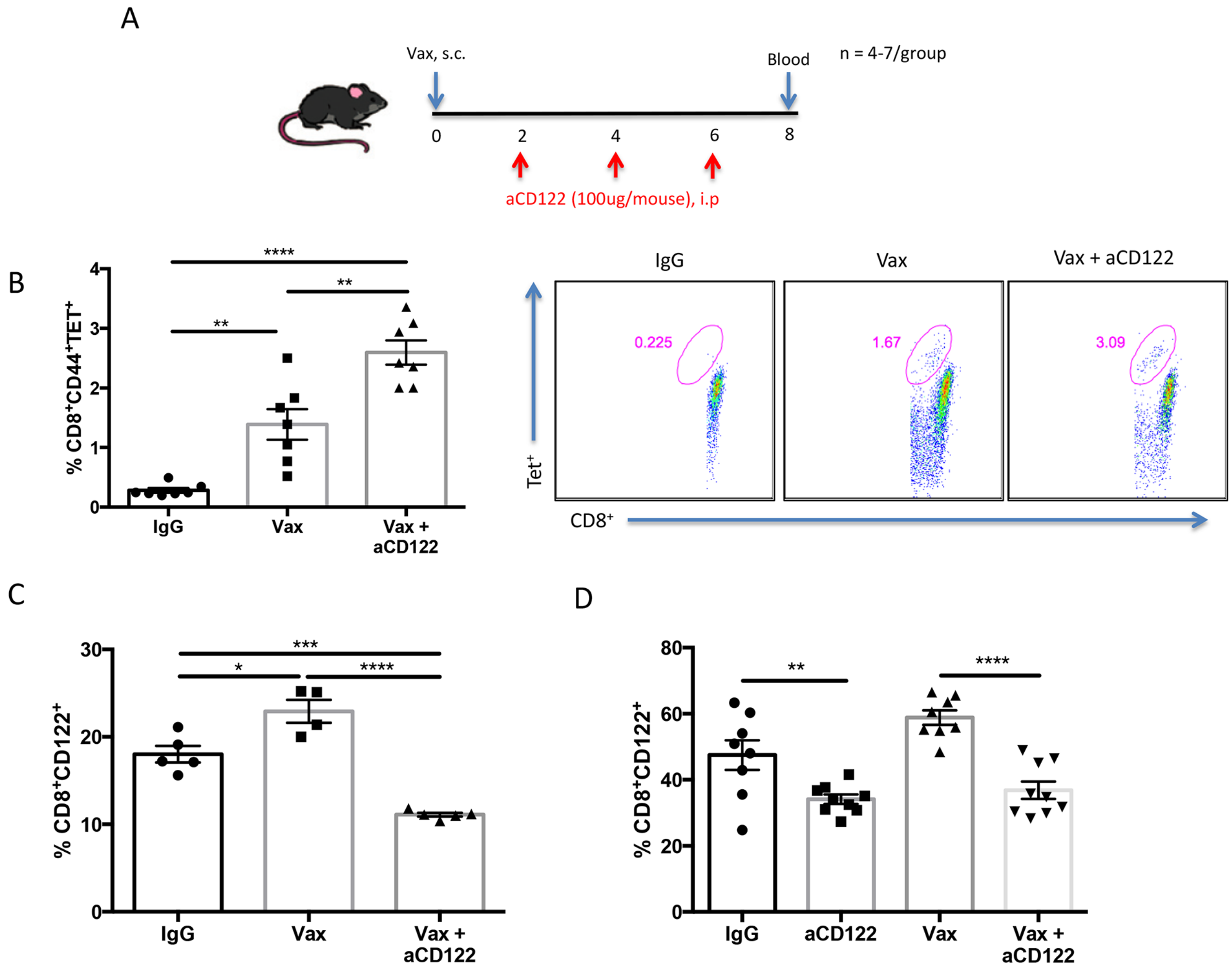

Supplementary Figure 2: aCD122 therapy enhanced vaccine-induced Ag-specific CD8^{+} T cell responses in the periphery of non-tumor-bearing mice. (A) Timeline of vaccine treatment (OVA257-264) and dosing schedule with or without aCD122 mAb treatment in mice. Immune responses were monitored at day 8 in the blood. **(B)** Representative scatter plot graph and dot plots show the percentages of H2-K^{b}-SIINFEKL-restricted OVA-specific CD8^{+} T cells. (**C & D**) Administration of aCD122 reduces the CD8^{+}CD122^{+} T cell population. (C) Scatter plot graph shows the percentage of CD8^{+}CD122^{+} T cells after aCD122 treatment in blood of mice. (D) Percentage of CD8+CD122+ T cells after therapy treatment in the tumors of mice from Figure 4B (16 days post tumor implantation). Results are representative of 3 independent experiments. *P<0.05; **P<0.01; ***P<0.001; ****P<0.0001. Errors bars indicate SEM; n = 4-8/group.
